# A robotic system for high-throughput automated lifespan and phenotyping analysis in C. elegans

**DOI:** 10.1093/geroni/igaa057.3270

**Published:** 2020-12-16

**Authors:** Elena Vayndorf, Jason Pitt, Judy Wu, Emily Chang, Richard Nguyen, Ashley Liang, Renee Ruan, Matt Kaeberlein

**Affiliations:** University of Washington, Seattle, Washington, United States

## Abstract

A goal of gerontology-related research is to develop therapies to improve the healthy period of life by understanding and targeting the molecular hallmarks of biological aging. Much progress has been made toward understanding the genetic and biochemical nature of these hallmarks through studies using simple invertebrate model organisms, such as the nematode Caenorhabditis elegans. Over the past decade, the identification of potential genetic and pharmacological modifiers of lifespan and age-related pathologies in C. elegans and other model organisms has yielded fruitful leads for follow-up investigation. However, such studies are typically time- consuming and labor-intensive. The goal of our work is to automate tasks that require frequent, repeated observations and hours of manual labor to collect and analyze lifespan, motility, and other behavioral data in C. elegans and other nematode models. The advent of affordable high-quality digital cameras, robotics systems, and 3D printers, as well as the decreasing financial and computational costs of image storage and processing, have allowed us to automate data capture and analysis on a large scale. To this end, our group recently developed a tool, we call the WormBot, consisting of an unbiased, high-throughput, automated robotic system and corresponding software, to perform genetic and pharmacological quantification of lifespan and health measures in C. elegans and related nematode species. We will report updates recently made to this system, including significant improvements to hardware, and present screening results from proteasome stimulator drugs known to reduce the accumulation of proteotoxic proteins linked to neurodegenerative diseases and aging.

